# No excess risk of death or multimorbidity following hemorrhagic stroke after mRNA vaccination compared with historical cases: a population-based cohort study

**DOI:** 10.1016/j.bbih.2026.101216

**Published:** 2026-03-17

**Authors:** Song Song, Yuqi Hu, Wenxin Tian, Cuiling Wei, Xinya Mu, Rachel Yui Ki Chu, Qi Sun, Yifang Huang, Zijie Xu, Wenlong Liu, Lingyue Zhou, Boyan Liu, Ian Chi Kei Wong, Francisco Tsz Tsun Lai

**Affiliations:** a Centre for Safe Medication Practice and Research, Department of Pharmacology and Pharmacy, Li Ka Shing Faculty of Medicine, The University of Hong Kong, Hong Kong Special Administrative Region of China; b Laboratory of Data Discovery for Health (D24H), Hong Kong Science Park, Sha Tin, Hong Kong Special Administrative Region of China; c Advanced Data Analytics for Medical Science (ADAMS) Limited, Hong Kong Special Administrative Region of China; d Department of Family Medicine and Primary Care, School of Clinical Medicine, Li Ka Shing Faculty of Medicine, The University of Hong Kong, Hong Kong Special Administrative Region of China; e Aston Pharmacy School, Aston University, Birmingham, United Kingdom; f National University of Singapore, Singapore, Singapore

**Keywords:** Adverse events of special interest, mRNA vaccine, Hemorrhagic stroke, Multimorbidity

## Abstract

**Introduction:**

While the association between hemorrhagic stroke and prior COVID-19 mRNA vaccination remains inconclusive, an examination of its prognosis may generate evidence on the potential causality of this relationship. If a causal link does exist, transient vaccine-related mechanisms (e.g., thrombocytopenia) might lead to a more favorable prognosis than naturally acquired cases.

**Aims:**

This study aimed to compare the prognosis of postvaccination hemorrhagic stroke and historical conventional cases with the same clinical diagnoses.

**Methods:**

A retrospective cohort study was conducted using a territory-wide electronic public healthcare database in Hong Kong, linked with population-based vaccination records. Since the roll-out of mRNA Vaccines (BNT162b2), patients aged 18 years or older hospitalized with hemorrhagic stroke within 28 days after mRNA vaccination were compared with conventional hemorrhagic stroke recorded between 2016 and 2017. The two-year follow-up period began from the diagnosis of hemorrhagic stroke. All-cause mortality and multimorbidity were examined using Cox proportional hazards models, with 95% confidence intervals (95%CIs) derived from bootstrap resampling (1000 iterations).

**Results:**

A total of 2578 patients were included for analysis: 110 in the postvaccination group and 2468 in the conventional group. Over the two-year follow-up period, all-cause death occurred in 27.27% (30/110) of the postvaccination group versus 29.78% (735/2468) in the conventional group. Multimorbidity was observed in 63.64% (70/110) of postvaccination cases and 73.14% (1805/2468) of conventional cases, respectively. Adjusted analyses showed no significant differences in all-cause mortality (adjusted Hazard Ratio [aHR] = 0.93, 95%CI:0.64-1.28) or multimorbidity risk (aHR = 0.85, 95%CI:0.66-1.05) between the two groups.

**Conclusion:**

Hemorrhagic stroke following mRNA vaccination had a similar long-term prognosis with conventional cases. These findings may suggest that most post-vaccination hemorrhagic strokes are coincidental rather than vaccine-induced and do not confer a different prognosis.

## 1 Introduction

The potential association between COVID-19 mRNA vaccination and the risk of hemorrhagic stroke remains inconclusive. Studies have suggested an increased risk of hemorrhagic stroke following mRNA vaccination (Patone et al., 2021; Chui et al., 2022; Dag Berild et al., 2022), while others have reported no significant change in risk (Ab et al., 2022; Botton et al., 2022; Lu et al., 2024), or even a reduced risk (Ihle-Hansen et al., 2023). A large-scale self-controlled case serious (SCCS) study in France (Botton et al., 2022), for instance, found no significant increase in hemorrhagic stroke within three weeks after either Pfizer-BioNTech or Moderna vaccination. However, an SCCS study (Patone et al., 2021) in England indicated that there was an increased risk of hemorrhagic stroke (IRR = 1.38, 95%CI: 1.12-1.71 at 15-21 days since vaccination) with BNT162b2. The differences among these studies highlight the need for a further examination.

Despite the lack of a consensus on causality, reports of hemorrhagic stroke occurring shortly after vaccination have raised public concerns. To date, most research has only focused on whether COVID-19 mRNA vaccination would trigger hemorrhagic stroke (Patone et al., 2021; Chui et al., 2022; Dag Berild et al., 2022; Ab et al., 2022; Botton et al., 2022; Lu et al., 2024; Ihle-Hansen et al., 2023). However, beyond the risk of occurrence, the clinical outcomes and long-term prognosis of hemorrhagic stroke following vaccination remain largely unexplored. It is unclear whether such cases differ in prognosis compared to conventional hemorrhagic stroke.

Insights from other vaccine-related adverse events suggest that prognosis may differ from naturally acquired cases. For instance, myocarditis, an established adverse effect of mRNA vaccines, has been shown to have a milder prognosis compared to conventional myocarditis (Lai et al., 2022a; Andrews et al., 2023; Husby et al., 2023). This difference could be partly explained by the mechanism of mRNA vaccines. The mechanism is that they contain mRNA encoding the spike protein of the virus, but do not introduce any whole live or dead virus, which means that there is no persistent viral agent to arouse a strong immune response against the heart muscle (Bozkurt et al., 2021). This suggests that vaccine-related conditions may follow distinct clinical courses. Several hypotheses have been proposed to explain a potential link between mRNA vaccination and hemorrhagic stroke (Ota et al., 2025; Cheda et al., 2024). One proposed mechanism involves vaccine-induced thrombocytopenia (Lee et al., 2021; Tarawneh and Tarawneh, 2021), which may result in localized and potentially less severe vascular injury. Therefore, we hypothesize that hemorrhagic stroke following mRNA vaccination may be associated with a more favorable prognosis than a broadly defined range of naturally acquired cases.

We conducted this population-based retrospective cohort study comparing the prognosis of postvaccination hemorrhagic stroke and historical conventional cases to further expand our knowledge on mRNA vaccine cardiovascular safety.

## 2 Methods

### 2.1 Study design and data sources

This retrospective cohort study utilized linked data from the Hospital Authority of Hong Kong and vaccination records maintained by the Department of Health. As the exclusive provider of public inpatient care and a major outpatient service provider, the Hospital Authority's database encompasses the entire Hong Kong population, given that all legal residents are eligible for public healthcare. All diagnoses in our database were made and entered by registered hospital doctors based on standard clinical work-up. This dataset has already been widely employed in pharmacovigilance research (Li et al., 2022a, 2022b; Lai et al., 2022b) due to its reliable diagnostic coding based on the International Classification of Diseases-9th Revision-Clinical Modification (ICD-9-CM).

### 2.2 Ethical approval

The study protocol received approval from the Institutional Review Board of the University of Hong Kong/Hospital Authority Hong Kong West (Ref: UW 23-329) and the Ethics Committee of the Department of Health (Ref: L/M 564/2023). Informed written consent has been waived by the ethics committees as this is an observational study using de-identified electronic health records. This study complies with the Declaration of Helsinki.

### 2.3 Cohort definition and follow-up

Starting from March 6, 2021, the date of rollout of the COVID-19 mRNA vaccine BNT162b2 in Hong Kong, individuals aged 18 and above diagnosed with hemorrhagic stroke (ICD-9-CM codes: 430.x, 431.x) within 28 days of any vaccine dose (initial, second, or booster) were identified for the postvaccination cohort. The identification period extended to April 30, 2022, allowing for a two-year follow-up, with data available until April 30, 2024. For comparison, hemorrhagic stroke diagnosed between January 1, 2016, and December 31, 2017, were included in the historical cohort, making sure that historical cases and their two-year follow up were neither related to COVID-19 nor mRNA vaccination. Only the first recorded episode was considered for individuals with multiple diagnoses. Patients were excluded if they had prior diagnoses of any subtype of stroke or multimorbidity, or positive COVID-19 polymerase chain reaction test result before the current episode. Follow-up commenced at the date of hemorrhagic stroke diagnosis and continued until the occurrence of an outcome, death (for non-mortality outcomes), or April 30, 2024, whichever occurred first.

### 2.4 Outcomes of interest

We assessed the incidence of all-cause death and multimorbidity within two years of hemorrhagic stroke diagnosis. Diagnostic codes for chronic conditions and multimorbidity are detailed in Table S1.

### 2.5 Covariates

Covariates included in multivariable models were age, sex, subtype of hemorrhagic stroke, history of hypertension, Charlson Comorbidity Index, health care utilization in the past year (number of hospitalizations), and history of medication. Medication use was treated as a binary composite variable and included anticoagulants, antiplatelets, and statins (see Table S2).

### 2.6 Statistical analysis

Standardized mean differences (SMDs) were used to compare baseline characteristics between groups. Kaplan-Meier estimators were applied to calculate cumulative incidence rates for each outcome, using time since diagnosis as the time scale. Adjusted cumulative incidences were estimated using the direct standardization method based on a multivariable Cox proportional hazards model, with 95% confidence intervals (CIs) derived from bootstrap resampling (1000 iterations).

Cox proportional hazards models were used to assess the associations between post-vaccination hemorrhagic stroke and subsequent outcomes. Adjusted hazard ratios (aHRs) and corresponding 95% CIs were calculated with covariate adjustment, using bootstrap resampling (1000 replications).

Subgroup analyses were performed by age (<60 and ≥ 60 years) and sex (male and female). Sensitivity analyses were conducted applying a Fine-Gray subdistribution hazard model to account for competing risks of all-cause death for multimorbidity, treating all-cause death as a competing event.

All statistical tests were two-sided, with significance defined as P < 0.05. Analyses were performed using R version 4.5.0.

## 3 Results

**Fig. 1:**
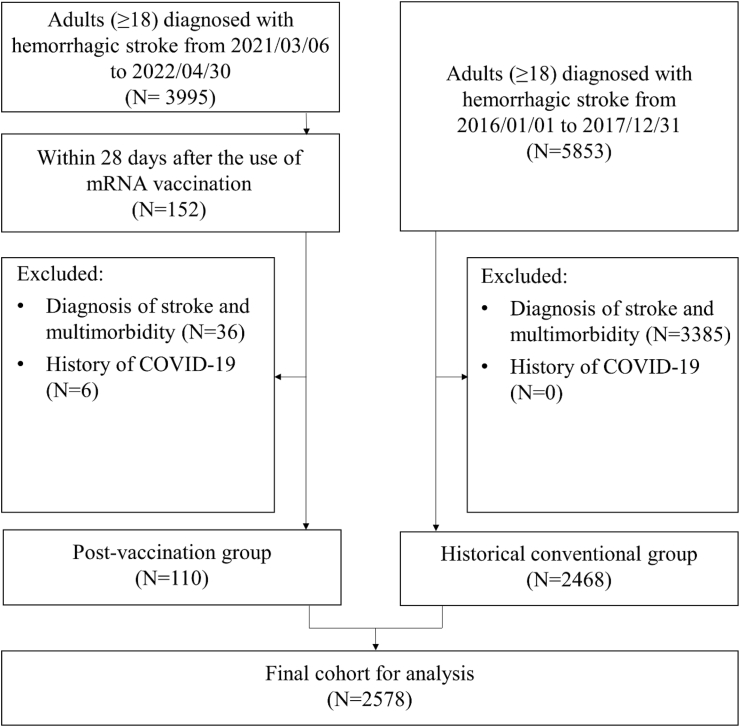
Flowchart of cohort selection.

By April 30, 2022, a total of 152 hemorrhagic stroke cases occurring within 28 days of mRNA vaccination were identified. Between 2016 and 2017, 5853 hemorrhagic stroke cases were recorded. After applying exclusion criteria, the final study population included 2578 individuals: 110 in the postvaccination group and 2468 in the historical group. The cohort selection process is illustrated in Fig. 1.

### 3.1 Baseline characteristics

Compared to the historical cohort, individuals with postvaccination hemorrhagic stroke were younger (mean age: 59.09 ± 14.32 vs. 61.97 ± 15.50 years) and more likely to be female (50.91% vs. 41.69%). The historical conventional group had higher rates of hypertension history and greater healthcare utilization. Detailed characteristics are presented in Table 1.

**Table 1:** Characteristics of historical conventional group and post-vaccination group.

Factors	Total	Historical conventional stroke	Postvaccine stroke	SMD
All	2578	2468	110	
Sex				0.186
Female	1085 (42.09)	1029 (41.69)	56 (50.91)	
Male	1493 (57.91)	1439 (58.31)	54 (49.09)	
Age at diagnosis, mean (SD), years	61.85 (15.46)	61.97 (15.50)	59.09 (14.32)	0.193
Subtype of hemorrhagic stroke				0.038
Subarachnoid hemorrhage	547 (21.22)	522 (21.15)	25 (22.73)	
Intracerebral hemorrhage	2031 (78.78)	1946 (78.85)	85 (77.27)	
History of medication^a^	328 (12.72)	311 (12.60)	17 (15.45)	0.082
Statin	221 (8.57)	207 (8.39)	14 (12.73)	0.142
Oral anticoagulants	34 (1.32)	31 (1.26)	3 (2.73)	0.105
Antiplatelets	216 (8.38)	208 (8.43)	8 (7.27)	0.043
History of hypertension				0.120
No	2093 (81.19)	1999 (81.00)	94 (85.45)	
Yes	485 (18.81)	469 (19.00)	16 (14.55)	
Charlson Comorbidity Index, mean (SD)	0.25 (0.70)	0.25 (0.71)	0.10 (0.41)	0.265
Hospitalization in the past year, times				0.105
0	2185 (84.76)	2088 (84.60)	97 (88.18)	
≥1	393 (15.24)	380 (15.40)	13 (11.82)	

Values are n (%) unless otherwise indicated.

SMD = Standardized mean difference.

a Composite binary indicator: some or none.

### 3.2 Primary outcomes

**Fig. 2:**
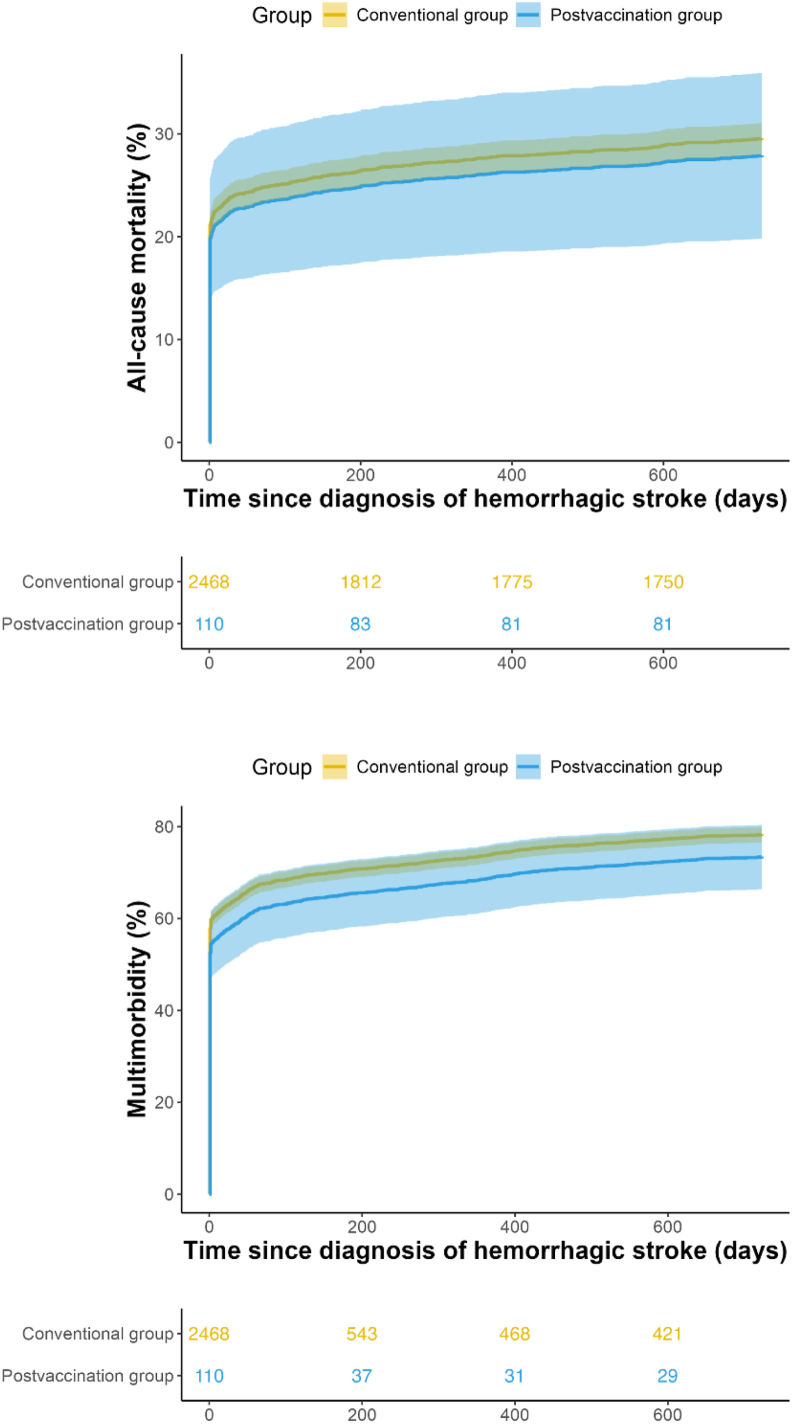
Adjusted cumulative incidences of death and multimorbidity during follow-up for patients with postvaccination stroke and patients with historical conventional stroke The adjusted cumulative incidences were estimated using the direct standardization method based on a multivariable Cox proportional hazards model (adjusted by age, sex, subtype of hemorrhagic stroke, history of hypertension, Charlson Comorbidity Index, prior health care utilization, and dichotomized history of medication), with 95% confidence intervals (CIs) derived from bootstrap resampling (1000 iterations).

Fig. 2 presented the adjusted cumulative incidence curves for all-cause mortality and multimorbidity over a two-year follow-up period, and Fig. S1 presented the crude cumulative incidence curves. No significant differences were observed between the post-vaccination and historical groups in Fig. 2.

Over the two-year follow-up period, all-cause death occurred in 27.27% (30/110) of the postvaccination group versus 29.78% (735/2468) in the historical group. Multimorbidity was observed in 63.64% (70/110) of postvaccination cases and 73.14% (1805/2468) of historical cases. Fig. S2 shows the proportions of chronic diseases within two years of follow-up from the diagnosis of hemorrhagic stroke. Adjusted analyses demonstrated no significant differences in all-cause mortality (aHR = 0.93, 95% CI: 0.64-1.28) or multimorbidity risk (aHR = 0.85, 95% CI: 0.66-1.05) between the two groups (Table 2, Table 3).

**Table 2:** Associations Between all-cause death and stroke groups over two years.

Population	Historical conventional stroke			Postvaccine stroke			HR (95%CI)^a^	
	Cohort size	No. of events (%)	Incidence rate^c^	Cohort size	No. of events (%)	Incidence rate^c^	Crude	Adjusted^b^
Main analysis								
All population	2468	735 (29.78)	204.99	110	30 (27.27)	181.24	0.83 (0.56-1.16)	0.93 (0.64-1.28)
Age group								
<60 years	1189	251 (21.11)	131.88	57	10 (17.54)	105.54	0.77 (0.34-1.24)	0.81 (0.35-1.33)
≥60 years	1279	484 (37.84)	287.69	53	20 (37.74)	282.57	0.90 (0.55-1.32)	1.05 (0.65-1.55)
Gender								
Male	1439	445 (30.92)	215.27	54	15 (27.78)	184.06	0.83 (0.46-1.24)	0.87 (0.49-1.32)
Female	1029	290 (28.18)	190.99	56	15 (26.79)	178.51	0.85 (0.47-1.30)	1.01 (0.57-1.51)

a Historical conventional stroke as reference group.

b Adjusted by age, sex, subtype of hemorrhagic stroke, history of hypertension, Charlson Comorbidity Index, prior health care utilization, and dichotomized history of medication (composite binary indicator).

c Incidence rates were the number of cases per 1000 person-years.

**Table 3:** Associations Between multimorbidity and stroke groups over two years.

Population	Historical conventional stroke			Postvaccine stroke			HR (95%CI)^a^	
	Cohort size	No. of events (%)	Incidence rate^c^	Cohort size	No. of events (%)	Incidence rate^c^	Crude	Adjusted^b^
Main analysis								
All population	2468	1805 (73.14)	1795.78	110	70 (63.64)	1044.43	0.75 (0.58-0.95)	0.85 (0.66-1.05)
Age group								
<60 years	1189	791 (66.53)	1209.52	57	30 (52.63)	629.74	0.67 (0.46-0.96)	0.77 (0.53-1.09)
≥60 years	1279	1014 (79.28)	2887.63	53	40 (75.47)	2063.6	0.86 (0.63-1.16)	0.93 (0.69-1.20)
Gender								
Male	1439	1101 (76.51)	2147.48	54	37 (68.52)	1241.95	0.78 (0.55-1.09)	0.89 (0.64-1.23)
Female	1029	704 (68.42)	1429.61	56	33 (58.93)	886.38	0.75 (0.51-1.03)	0.80 (0.57-1.09)

a Historical conventional stroke as reference group.

b Adjusted by age, sex, subtype of hemorrhagic stroke, history of hypertension, Charlson Comorbidity Index, prior health care utilization, and dichotomized history of medication (composite binary indicator).

c Incidence rates were the number of cases per 1000 person-years.

Subgroup analyses (stratified by age and sex) and sensitivity analyses (Table S3) showed results consistent with the main analysis.

## 4 Discussion

This territory-wide real-world study in Hong Kong found no significant difference in all-cause mortality or multimorbidity between patients with hemorrhagic stroke occurring after COVID-19 mRNA vaccination and those with historical conventional hemorrhagic stroke. The subgroup analyses and sensitivity analyses showed that our results are robust. Our study suggested postvaccination hemorrhagic stroke did not confer worse prognosis compared to historical conventional hemorrhagic stroke.

Existing research has mostly focused on the risk of hemorrhagic stroke after vaccination rather than prognosis of postvaccination hemorrhagic stroke. Some large-scale epidemiological studies and meta-analyses have shown no increased risk of hemorrhagic stroke following COVID-19 vaccination. A comprehensive systematic review and meta-analysis (Liu et al., 2023) encompassing observational studies and nearly 80 million participants revealed no overall increase in hemorrhagic stroke risk after vaccination when assessed in cohort studies (IRR = 0.75, 95% CI: 0.67-0.85). This is further supported by studies from Norway (Ihle-Hansen et al., 2023) and other populations (Ab et al., 2022; Botton et al., 2022; Lu et al., 2024), which have consistently shown no elevated risk of hemorrhagic stroke after mRNA vaccination. A nationwide time-series correlation study conducted in Korea (Hyeon Cho et al., 2024) showed that the incidence change of hemorrhagic stroke during the pandemic was associated with conventional risk factors but not with SARS-CoV-2 infection or vaccination. Our study extends this evidence by demonstrating that, among individuals with hemorrhagic stroke after vaccination, the incidences of all-cause mortality and multimorbidity were highly comparable to those of conventional cases.

Several hypotheses have been proposed to explain a potential link between mRNA vaccination and hemorrhagic stroke, like immune-induced thrombocytopenia, vascular injury, and spike protein-related endothelial effects (Ota et al., 2025; Cheda et al., 2024). The spike protein encoded by mRNA vaccines may disrupt blood-brain barrier integrity, potentially contributing to neurological complications in patients (Ota et al., 2025). If such mechanisms were clinically relevant on a population level, one might expect vaccine-related hemorrhagic strokes to present with a different, milder, prognosis. However, we did not observe any prognostic difference in terms of mortality or multimorbidity incidence among patients with post-vaccination hemorrhagic stroke comparing with those with conventional hemorrhagic stroke. This suggests that most hemorrhagic strokes occurring after mRNA vaccination are likely coincidental events driven by underlying conventional risk factors rather than direct vaccine-induced pathology. Alternatively, if the association were indeed causal, the proportion of actual vaccine-induced cases would have been extremely small, which implies the extreme rarity of it as a side effect.

Our results have important clinical and public health implications. Our findings support the standard evidence-based management for post-vaccination hemorrhagic stroke. Our findings also provide reassurance regarding vaccine safety. Most hemorrhagic strokes reported following mRNA vaccination are most likely incidental occurrences attributable to pre-existing conventional vascular risk factors, rather than a direct vaccine-related pathogenic mechanism. This supports the ongoing and future widespread use of mRNA vaccines (May 2024; Lorentzen et al., 2022). When evaluating suspected vaccine-related adverse events, clinicians should consider the role of prior health status and the possibility of coincidental timing.

There are clear strengths to the current study. First, this is the first study comparing the prognosis of hemorrhagic stroke after mRNA vaccination with conventional hemorrhagic stroke. We made use of comprehensive clinical data from a large sample with a follow-up period of up to two years. Second, all diagnoses in our database were made and entered by registered hospital doctors based on standard clinical work-up, with previous validation studies suggesting high accuracy of the data (Wong et al., 2016; Lau et al., 2017). Third, the selection of a historical cohort of hemorrhagic stroke from 2016 to 2017 ensured that the cases and their follow up were neither related to COVID-19 nor mRNA vaccination.

Several limitations warrant consideration. First, we could not validate the diagnoses with anonymized clinical investigative data, which are unavailable in our database (Cunningham et al., 2006). However, a previous high-quality pharmacovigilance study (Wong et al., 2016) have demonstrated a high diagnostic coding accuracy of our database. For instance, the overall positive predictive value for stroke exceeded 90%. Second, because the event rate of some of the outcomes was low, we could only include a limited number of covariates in the model, and medications were operationalized as some vs none. Third, the historical comparison cannot separate the effect of exposure from changes in medical care and baseline risk over time. Even though we adjusted for measurable factors and ran multiple sensitivity analyses, some bias related to calendar time may remain. Finally, the rare incidence number of postvaccination hemorrhagic stroke also limited our sample size and numbers of events, resulting in substantially wide CIs for some estimates. To enhance robustness, we performed bootstrap-based confidence intervals, stratified analyses, and sensitivity analyses, all of which were directionally consistent.

This study demonstrated that hemorrhagic stroke following mRNA vaccination had a long-term prognosis like conventional cases. These findings suggested that most post-vaccination hemorrhagic strokes are coincidental rather than vaccine-induced and do not confer worse prognosis, reinforcing the safety of mRNA vaccine.

## CRediT authorship contribution statement

**Song Song:** Writing – review & editing, Writing – original draft, Software, Methodology, Investigation, Formal analysis, Data curation, Conceptualization. **Yuqi Hu:** Writing – review & editing. **Wenxin Tian:** Writing – review & editing. **Cuiling Wei:** Writing – review & editing. **Xinya Mu:** Writing – review & editing. **Rachel Yui Ki Chu:** Writing – review & editing. **Qi Sun:** Writing – review & editing. **Yifang Huang:** Writing – review & editing. **Zijie Xu:** Writing – review & editing. **Wenlong Liu:** Writing – review & editing. **Lingyue Zhou:** Writing – review & editing. **Boyan Liu:** Writing – review & editing. **Ian Chi Kei Wong:** Writing – review & editing, Conceptualization. **Francisco Tsz Tsun Lai:** Writing – review & editing, Writing – original draft, Supervision, Funding acquisition, Conceptualization.

## Disclosures

ICKW received research grants from Amgen, Janssen, GSK, Novartis, Pfizer, Bayer and Bristol-Myers Squibb and Takeda, Institute for Health Research in England, European Commission, National Health and Medical Research Council in Australia, The European Unions Seventh Framework Programme for research, technological development, Research Grants Council Hong Kong and Health and Medical Research Fund Hong Kong; consulting fees from IQVIA and World Health Organization; payment for expert testimony for Appeal Court in Hong Kong; serves on advisory committees for Member of Pharmacy and Poisons Board; is a member of the Expert Committee on Clinical Events Assessment Following COVID-19 Immunization; is a member of the Advisory Panel on COVID-19 Vaccines of the Hong Kong Government; is the non-executive director of Jacobson Medical in Hong Kong; and is the founder and director of Therakind Limited (UK), Advance Data Analytics for Medical Science (ADAMS) Limited (HK), Asia Medicine Regulatory Affairs (AMERA) Services Limited and OCUS Innovation Limited (HK, Ireland and UK). FTTL was supported by the RGC Postdoctoral Fellowship under the Education Bureau of the Hong Kong Special Administrative Region Government and has received research grants from the Health Bureau as well. The other authors declare no competing interest.

## Clinical trial number

Not applicable.

## Funding

The project was supported by the Health and Medical Research Fund under the Health Bureau of Hong Kong (Ref No. COVID19F01, 23221112).

## Declaration of competing interest

ICKW received research grants from Amgen, Janssen, GSK, Novartis, Pfizer, Bayer and Bristol-Myers Squibb and Takeda, Institute for Health Research in England, European Commission, National Health and Medical Research Council in Australia, The European Unions Seventh Framework Programme for research, technological development, Research Grants Council Hong Kong and Health and Medical Research Fund Hong Kong; consulting fees from IQVIA and World Health Organization; payment for expert testimony for Appeal Court in Hong Kong; serves on advisory committees for Member of Pharmacy and Poisons Board; is a member of the Expert Committee on Clinical Events Assessment Following COVID-19 Immunization; is a member of the Advisory Panel on COVID-19 Vaccines of the Hong Kong Government; is the non-executive director of Jacobson Medical in Hong Kong; and is the founder and director of Therakind Limited (UK), Advance Data Analytics for Medical Science (ADAMS) Limited (HK), Asia Medicine Regulatory Affairs (AMERA) Services Limited and OCUS Innovation Limited (HK, Ireland and UK). FTTL was supported by the RGC Postdoctoral Fellowship under the Education Bureau of the Hong Kong Special Administrative Region Government and has received research grants from the Health Bureau as well. The other authors declare no competing interest.

## Data Availability

Data used for this study will not be available to others as the data custodians have not given permission due to concerns over patient privacy protection. Requests for data access could be submitted to the Central Panel on Administrative Assessment of External Data Requests of the Hospital Authority (hacpaaedr@ha.org.hk). As the data provided will be customized for the specific purpose of each project, the time duration required to process such requests may vary. Upon data request approval, no sharing of such data with third parties is allowed.
