# Supplementary material for: No excess risk of death or multimorbidity following hemorrhagic stroke after mRNA vaccination compared with historical cases: a population-based cohort study
Source: Brain Behav Immun Health. 2026 Mar 17;53:101216. doi: 10.1016/j.bbih.2026.101216 (PMC13018963; doi:10.1016/j.bbih.2026.101216)
Supplement: Multimedia component 1 [file mmc1.pdf]

Table S1. Diagnostic codes used to operationalize chronic conditions and multimorbidity

Chronic conditions	International Classification of Diseases, Ninth Revision	International Classification of Primary Care
Alcohol misuse	265.2, 291.1–291.3, 291.5–291.9, 303.0, 303.9, 305.0, 357.5, 425.5, 535.3, 571.0–571.3, 980, V11.3	P15
Asthma	493	R96
Atrial fibrillation	427.3	K78
Cancer, lymphoma	200–202, 203.0, 238.6	B72
Cancer, metastatic	196–199	B74, D74, D76, D77, L71, N74, S77, T71, U75, U76, U77, W72, X77, Y78
Cancer, non-metastatic (breast, cervical, colorectal, lung, prostate)	153-154, 162-163, 174, 180, 185, 230.3-230.6, 231.2, 233.0-233.1, 233.4	D75, R84, X75, X76, Y77
Chronic heart failure	398.91, 402.01, 402.11, 402.91, 404.01, 404.03, 404.11, 404.13, 404.91, 404.93, 428	K77
Chronic kidney disease	583, 584, 585, 586, 592, 593.9	U14
Chronic pain	307.80, 307.89, 338.0, 338.2, 338.4, 719.41, 719.45 - 719.47, 719.49, 720.0, 720.2, 720.9, 721.0 - 721.4, 721.6, 721.8, 721.9, 722, 723.0, 723.1, 723.3 - 723.9, 724.0 - 724.6, 724.70, 724.79, 724.8, 724.9, 729.0 - 729.2, 729.4, 729.5	A01
Chronic pulmonary disease	416.8, 416.9, 490–492, 494-505, 506.4, 508.1, 508.8	R95
Chronic viral hepatitis B	70.2-70.3	D72
Cirrhosis	571.2, 571.5, 571.6, 456.0, 456.1, 456.20, 456.21, 567.0, 567.2, 567.21, 567.29, 567.8,	-

	567.9, 572.2, 572.4, 789.5 (Exclude 567.81, 567.82, 789.51)	
Dementia	290, 294.1, 331.2	P70
Depression	296.2, 296.3, 296.5, 300.4, 309, 311	P76
Diabetes	250	T89-T90
Epilepsy	345	N88
Hypertension	401-405	K86-K87
Hypothyroidism	240.9, 243, 244, 246.1, 246.8	T86
Inflammatory bowel disease	555, 556	-
Irritable bowel syndrome	564.1 (Exclude 153-154, 157, 183.0, 197.5, 198.6, 235.2, 239.0, 555- 556, 571.2, 571.5, 577.1, 579)	D93
Multiple sclerosis	323, 340, 341.0, 341.9, 377.3	N86
Myocardial infarction	410	K75
Parkinson’s disease	332	N87
Peptic ulcer disease	531.7, 531.9, 532.7, 532.9, 533.7, 533.9, 534.7, 534.9	D86
Peripheral vascular disease	440.2	K92
Psoriasis	696.1	S91
Rheumatoid arthritis	446.5, 710.0–710.4, 714.0–714.2, 714.8, 725	L88
Schizophrenia	295	P72
Severe constipation	560.1, 560.30, 560.39, 560.9, 564.0, 569.83, 569.89 (Exclude 152-154, 158, 179-189, 197.5-197.6, 235.2, 239.0, 555-556, 568.0, 614.6, (560.9 if 789.01, 789.02, 789.06))	D12
Stroke	430, 431, 433.x1, 434.x1, 436	K90

Table S2. Drug name and British National Formulary (BNF) code used to identify prescriptions.

Description	BNF
Statin	2.12
Anticoagulant	2.8.2
Antiplatelet medications	2.9

Table S3 Associations Between multimorbidity and stroke groups over two years based on competing risk model

Population	Historical conventional stroke		Postvaccine stroke		sHR (95%CI) [a]	
	Cohort size	No. of events (%)	Cohort size	No. of events (%)	Crude	Adjusted [b]
Main analysis						
All population	2468	1805 (73.14)	110	70 (63.64)	0.82 (0.66-0.99)	0.89 (0.72-1.05)
Age group						
<60 years	1189	791 (66.53)	57	30 (52.63)	0.73 (0.52-0.99)	0.80 (0.58-1.07)
≥60 years	1279	1014 (79.28)	53	40 (75.47)	0.93 (0.72-1.14)	0.95 (0.76-1.14)
Gender						
Male	1439	1101 (76.51)	54	37 (68.52)	0.85 (0.64-1.08)	0.92 (0.71-1.16)
Female	1029	704 (68.42)	56	33 (58.93)	0.82 (0.60-1.05)	0.85 (0.64-1.09)

[a] Historical conventional stroke as reference group.

[b] Adjusted by age, sex, subtype of hemorrhagic stroke, history of hypertension, Charlson Comorbidity Index, prior health care utilization, and dichotomized history of medication (composite binary indicator).

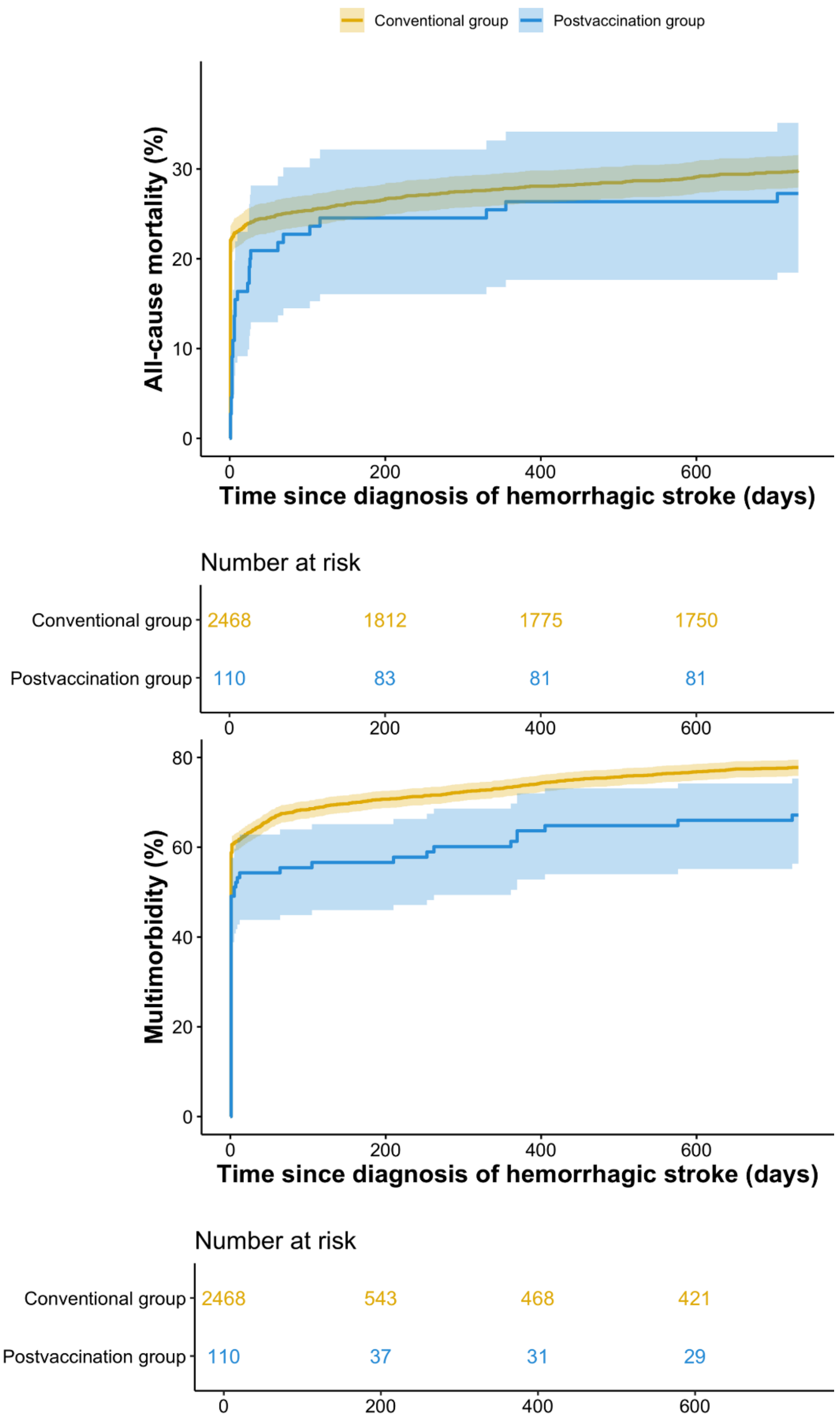

Figure S1 Crude cumulative incidences of death and multimorbidity during follow-up for patients with postvaccination stroke and patients with historical conventional stroke

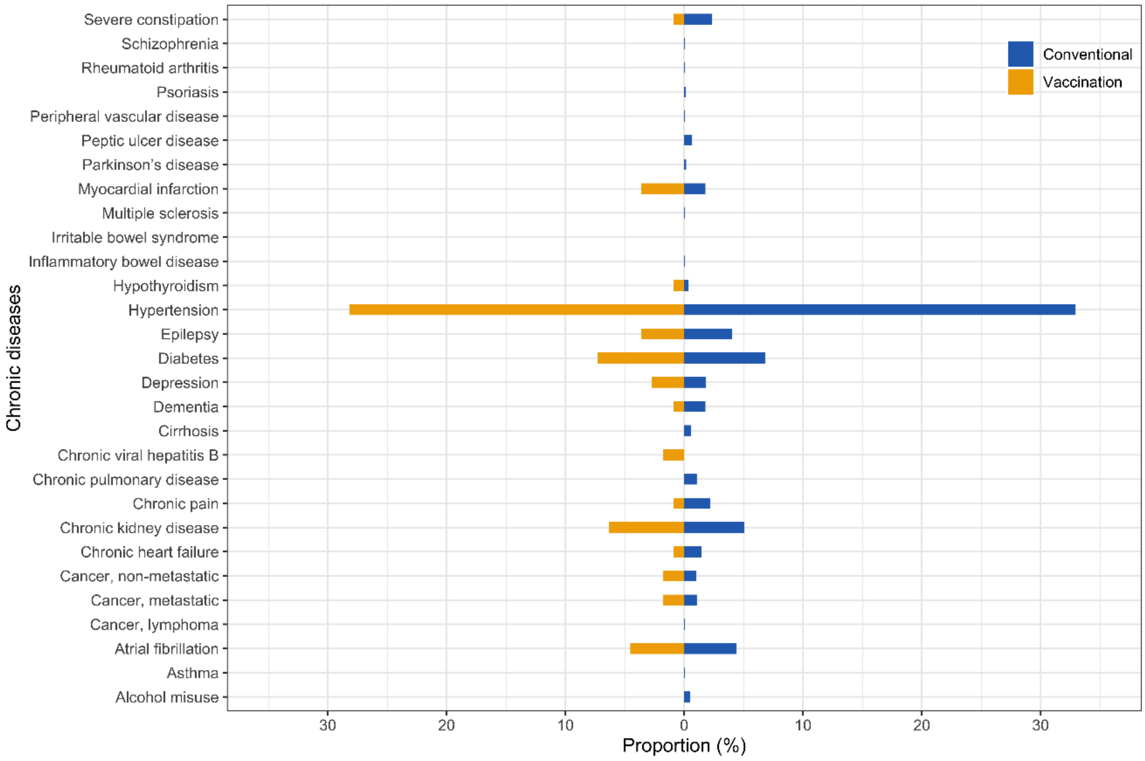

Figure S2 Proportions of chronic diseases within two years of follow-up from the diagnosis of hemorrhagic stroke
